# Three-Dimensional Compaction Switches Stress Response Programs and Enhances Therapeutic Efficacy of Endometrial Mesenchymal Stem/Stromal Cells

**DOI:** 10.3389/fcell.2020.00473

**Published:** 2020-06-16

**Authors:** Alisa Domnina, Julia Ivanova, Larisa Alekseenko, Irina Kozhukharova, Aleksandra Borodkina, Natalia Pugovkina, Irina Smirnova, Olga Lyublinskaya, Irina Fridlyanskaya, Nikolay Nikolsky

**Affiliations:** Department of Intracellular Signaling and Transport, Institute of Cytology of the Russian Academy of Sciences, Saint Petersburg, Russia

**Keywords:** mesenchymal stem cells, endometrial stem/stromal cells, spheroids, therapeutic potential, oxidative stress, heat shock, apoptosis, stress response

## Abstract

Mesenchymal stem cells are currently tested as a promising tool for the treatment of a wide range of human diseases. Enhanced therapeutic potential of spheroids formed from these cells has been proved in numerous studies, however, the fundamental basics of this effect are still being discussed. In this work, we showed that endometrial mesenchymal stem/stromal cells (eMSCs) assembled in spheroids possess a higher therapeutic efficacy compared to cells grown in monolayer in the treatment of the defects that are non-specific for eMSC tissue origin – skin wounds. With the purpose to elucidate the possible causes of superior spheroid potency, we compared the tolerance of eMSC cultivated in spheres and monolayer to the stress insults. Using genetically encoded hydrogen peroxide biosensor HyPer, we showed that three-dimensional configuration (3D) helped to shield the inner cell layers of spheroid from the external H_2_O_2_-induced oxidative stress. However, the viability of oxidatively damaged eMSCs in spheroids appeared to be much lower than that of monolayer cells. An extensive analysis, which included administration of heat shock and irradiation stress, revealed that cells in spheroids damaged by stress factors activate the apoptosis program, while in monolayer cells stress-induced premature senescence is developed. We found that basal down-regulation of anti-apoptotic and autophagy-related genes provides the possible molecular basis of the high commitment of eMSCs cultured in 3D to apoptosis. We conclude that predisposition to apoptosis provides the programmed elimination of damaged cells and contributes to the transplant safety of spheroids. In addition, to investigate the role of paracrine secretion in the wound healing potency of spheroids, we exploited the *in vitro* wound model (scratch assay) and found that culture medium conditioned by eMSC spheroids accelerates the migration of adherent cells. We showed that 3D eMSCs upregulate transcriptional activator, hypoxia-inducible factor (HIF)-1, and secret ten-fold more HIF-1-inducible pro-angiogenic factor VEGF (vascular endothelial growth factor) than monolayer cells. Taken together, these findings indicate that enhanced secretory activity can promote wound healing potential of eMSC spheroids and that cultivation in the 3D cell environment alters eMSC vital programs and therapeutic efficacy.

## Introduction

Mesenchymal stem/stromal cells (MSCs) are the subject of numerous fundamental and applied research due to their unique biological properties and high therapeutic potential, proven by clinical trials (Bernardo et al., 2012; Fitzsimmons et al., 2018; Hoogduijn and Lombardo, 2019). Initially, in the mid-60s of the 20th century, MSCs were isolated from bone marrow (Friedenstein et al., 1970). Further, this type of cells was found in almost all connective tissues of the body – adipose tissue, umbilical blood, placenta, amniotic fluid, dental pulp, and endometrium (Andrzejewska et al., 2019). According to the criteria established by the International Society for Cellular Therapy (Dominici et al., 2006), cultured MSCs express surface markers CD90, CD105, and CD73, but do not show hematopoietic markers expression; possess the high adhesion capacity to plastic surfaces, fibroblast-like morphology, as well as ability to differentiate into adipocytes, chondrocytes and osteoblasts. Due to their immunomodulatory and pro-angiogenic properties proven in patients (Petinati et al., 2018), MSCs are currently considered to be an effective tool for the treatment of degenerative, autoimmune, and inflammatory diseases, as well as injuries and strokes (Wang et al., 2012). The majority of clinical trials use the cellular suspension of MSCs as the therapeutic agent. However, the latest research suggests putting into practice the MSCs, incorporated into various three-dimensional (3D) and tissue-like structures (Baptista et al., 2018; Nimiritsky et al., 2019; Yao et al., 2020). The imitation of tissue-like cell organization *in vitro* has become possible with the development of 3D models of cell growth, such as scaffolds based on different synthetic or natural materials and seeded with cells, as well as scaffold-free models – cell spheroids (Han et al., 2019).

Spheroids, originally emerged as 3D aggregates of tumor cells, have long been used in cell biology as a model for studying the hierarchical structure of tumors and their microenvironment, as well as for testing various antitumor drugs (Sant and Johnston, 2017). Later on, this model of cell growth has become applicable for the cultivation of MSCs isolated from different tissues (Bartosh et al., 2010; Baraniak and McDevitt, 2012; Lee et al., 2016; Cui et al., 2017; Domnina et al., 2018). When culturing in 3D configuration the plasticity of MSCs leads to the phenotype shifts and acquirement of the features unusual for their two-dimensional (2D) cultures (Yeh et al., 2014; Forte et al., 2017; Han et al., 2019). For instance, generation of the hypoxic zone in the center of spheroid causes the expression of hypoxia-associated genes, such as the key transcription factor induced by hypoxia, HIF-1 (hypoxia-inducible factor 1), which enhances the synthesis of pro-survival proteins and increase the adaptive abilities of cells. Cultivation in spheroids augmentes the angiogenic potential of MSCs due to increased secretion of growth factors (VEGF, HGF, and FGF2), enhances anti-inflammatory and anti-apoptotic MSC properties due to the upregulation of such genes as TSG-6 (TNFα-induced gene/protein 6), STC-1 (staniocalcin-1), and PGE2 (prostaglandin E2; Bartosh et al., 2010; Madrigal et al., 2014; Lee et al., 2016; Murphy et al., 2017). In addition, 3D MSC substantially enhance secretion of chemokines and cytokines, as well as expression of their receptors, such as CXCR4 (CXC chemokine receptor 4) and CMKLR1 (chemokine-like receptor 1) that stimulate their immunomodulatory and “homing” capacities (Zhang et al., 2012; Madrigal et al., 2014).

Changes in the molecular and functional properties of MSCs cultivated in spheroids open up the new prospects for the clinical use of these cells. Currently, numerous preclinical studies with the use of MSC spheres are conducted, aimed at the correction of various human diseases, such as skeletal system diseases, ischemic and cardiovascular disorders and wound healing (Wang et al., 2009; Amos et al., 2010; Bhang et al., 2012; Zhang et al., 2012; Emmert et al., 2013). We have previously demonstrated that transplantation of spheroids from human endometrial MSCs (eMSCs) can be used in the treatment of infertility (Domnina et al., 2018). Using a model of Asherman’s syndrome in rats (a model of infertility caused by replacement of the normal endometrium with connective tissue as a result of damage), we showed that the intrauterine administration of eMSCs in spheroids results in a better therapeutic effect than the administration of eMSCs after cultivation in a monolayer. The therapeutic effect was assessed by the offspring number and by the number of offspring pregnancy in model animals (Domnina et al., 2018). In the current study, we use eMSC spheroids to heal the defects that are non-specific for eMSC tissue origin – skin wounds. We show that spheroid transplantation stimulates wound healing in experimental animals more efficiently than transplanting a suspension of cells cultured in a monolayer, and discuss the possible causes of the enhanced therapeutic potential of 3D eMSCs.

## Results

### eMSC Spheroid Transplantation Stimulates Skin Wound Healing

**FIGURE 1:**
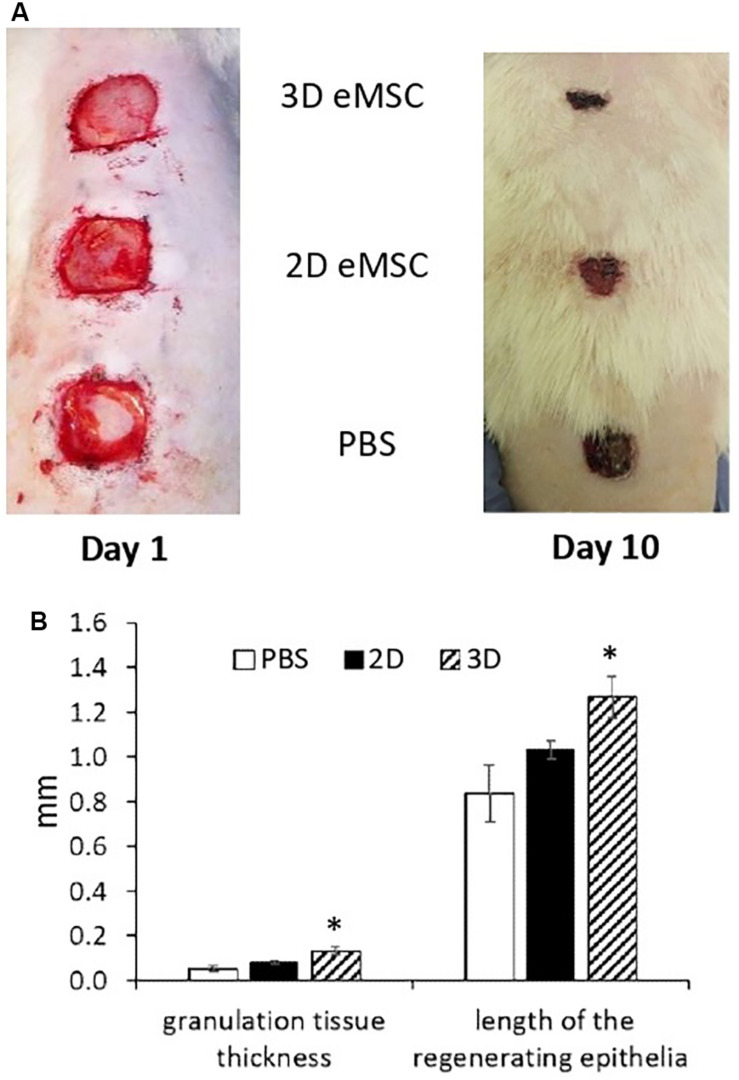
Wound closure in rat after 2D and 3D eMSC transplantation. **(A)** Day 1 (left image) and 10 (right image) after eMSC (passage 7) transplantation. **(B)** Granulation tissue thickness and length of the regenerating epithelia in the rat wound on the seventh day after eMSC transplantation. Data are shown as mean ± SD. Two-tailed Student’s *t*-test was utilized for pairwise comparison. ^∗^*p* < 0.05 vs. PBS and 2D eMSCs.

We evaluated the effect of 3D eMSC transplantation on the wound healing process of experimental animals (rats). In these experiments, pieces of full-thickness skin (1.5 cm × 1.5 cm) on the rat back were excised to create a skin wounds (Figure 1A). Immediately after, monolayer eMSC in suspension (4 × 10^6^ cells/per wound) and eMSC spheroids (4 × 10^6^ cells in total/per wound) were injected in PBS solution around the prepared full-thickness skin wounds. The administration of PBS was used as a negative control. On the day of surgery and every day thereafter the open wounds were photographed and the reduction of the wound size was analyzed (Supplementary Figure S1A). On day 10 (see Figure 1A), the rate of the wound closure was as follows: the wounds treated with eMSC spheroids were almost closed (85 ± 3%); the wounds treated with monolayer eMSCs were partly closed with new tissue (77 ± 2%); in control animals, where PBS solution was administered, wound closure was low (57 ± 4%). Moreover, on day 7 after transplantation, the thickness of granulation tissue and the length of the regenerating epithelium in histological sections were higher in the case of eMSC spheroids application compared to the control wounds (see Figure 1B and Supplementary Figure S1B). Complete wound healing was observed: on day 12 – for eMSC spheroids, on day 14 – for eMSC, cultivated in monolayer, on day 16 – for non-treated wounds (PBS).

The results presented in this section showed that eMSC spheroids can be applied to stimulate skin regeneration and that 3D eMSCs potentiate wound healing more effectively than 2D eMSCs. Next, we attempted to figure out which properties determine the high regenerative potential of spheroids.

### Outer Cell Layers Protect a Spheroid Core From Oxidative Stress

The protection of inner cell layers in spheroids from the harmful impacts during transplantation is often considered as one of the possible causes of their high therapeutic potential.

Since the processes of tissue regeneration, including wound healing, are often accompanied by the local oxidative stress, we analyzed the response of cells cultivated in spheroids to H_2_O_2_-induced oxidative damage. We used eMSC line with stable expression of genetically encoded hydrogen peroxide biosensor HyPer-cyto (Belousov et al., 2006). This sensor is localized in cell cytosol and its ratiometric fluorescence signal is used as a measure of intracellular peroxide concentration.

**FIGURE 2:**
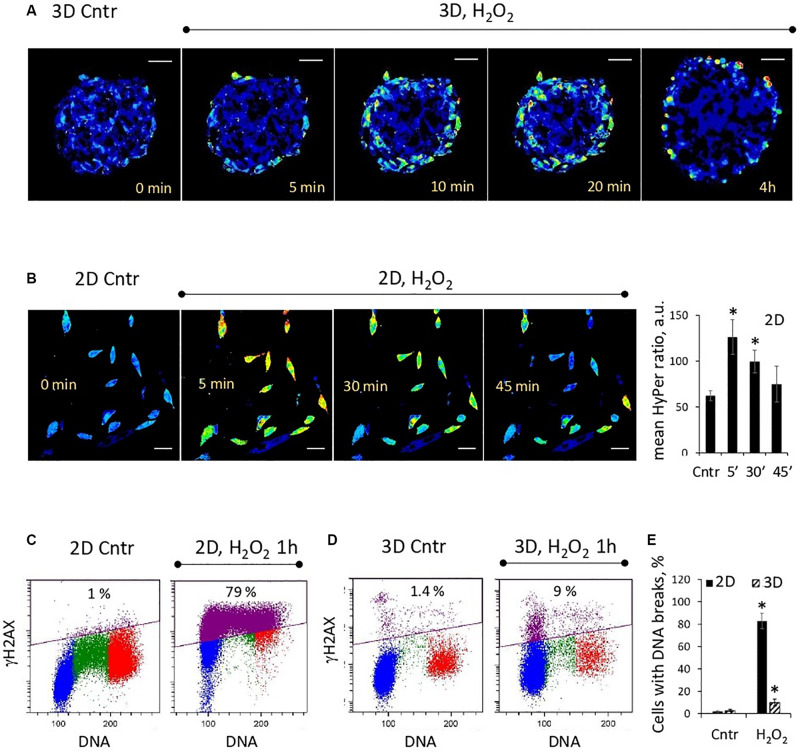
Outer cell layers protect the inner cell mass of the spheroid from the damaging effects of oxidative stress. **(A)** Weak penetration of H_2_O_2_ inside the spheroids: ratiometric images of HyPer-expressing spheroids just after adding H_2_O_2_ (200 μM) to the cell medium. **(B)** Dynamics of H_2_O_2_ consumption by monolayer cells (passage 11): ratiometric images of HyPer-expressing cells just after adding H_2_O_2_ (200 μM) to the cell medium (left) and quantification of the mean HyPer ratio signal per cell (right). Data are shown as mean ± SD (*N* = 15). ^∗^*p* < 0.05 vs. the control values. **(C,D)** Flow cytometry assay for DNA breaks in 2D and 3D eMSCs (passage 12): test for γH2AX-positive cells, performed after 1-h exposition to 200 μM of H_2_O_2_. Blue, green and red markings correspond to cells in the G_0_/G_1_, S, and G_2_/M phases of the cell cycle, respectively. Violet color marks γH2AX-positive cells. **(E)** Fraction of γH2AX-positive cells in 2D and 3D eMSCs treated with 200 μM of H_2_O_2_ for 1 h. Data are shown as mean ± SD (*N* = 3). ^∗^*p* < 0.05 vs. the control values. Scale bar: 50 μm. Cntr, control cells.

Analysis of the dynamic changes in the HyPer fluorescence signal after addition of H_2_O_2_ (200 μM) to the spheroid culture medium showed that peroxide permeated only the outer layers of spheroids (Figure 2A). After 4 h of incubation, H_2_O_2_ was completely metabolized by the outer cells, and the HyPer signal returned to its basal level. In contrast to 3D eMSCs, cells in the monolayer culture, exposed to the same concentration of H_2_O_2_, were all subjected to peroxide oxidation (Figure 2B). DNA integrity test, carried out 1 h after H_2_O_2_ exposure revealed much more cells (83 ± 7% of total cell population) with histone H2AX phosphorylation foci (γH2AX foci) in the eMSC monolayer in comparison with cells, cultured in 3D (10 ± 3% of total cell population) (Figures 2C–E). This data confirm the shielding effect of the outer cell layers against the hydrogen peroxide damage in spheroids.

### 3D eMSCs Respond to Stress by Activating the Apoptosis Program

**FIGURE 3:**
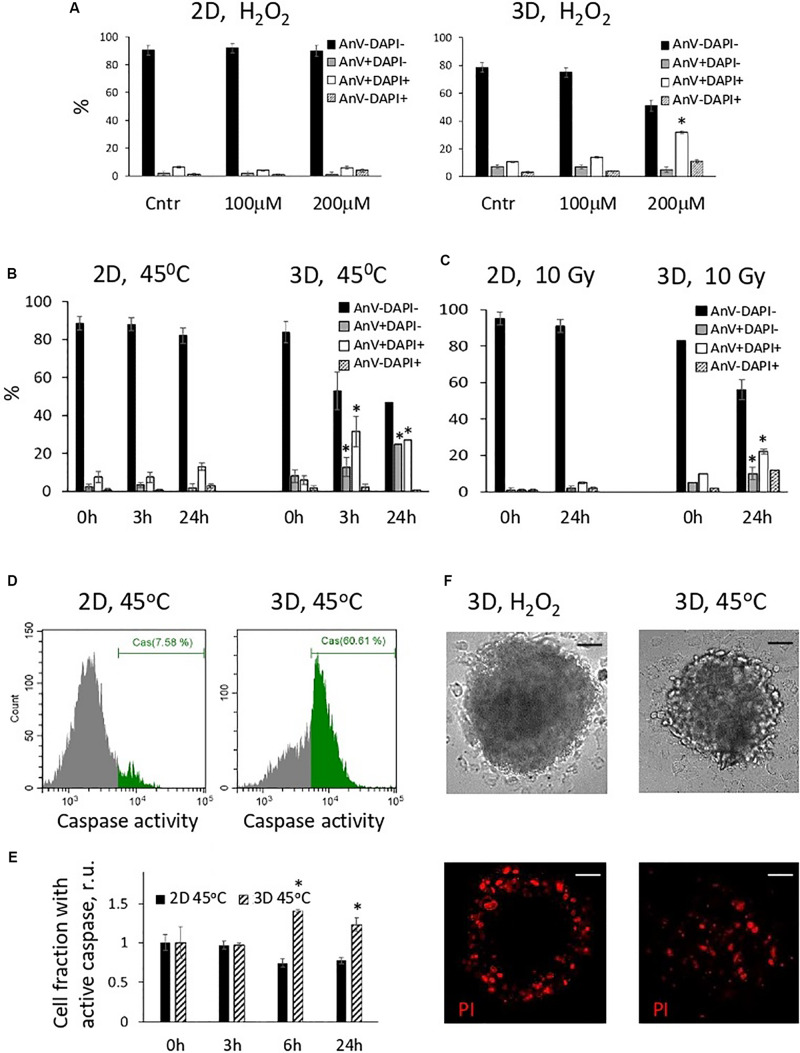
Cells in spheroids, in contrast to monolayer eMSCs, activate apoptotic program in response to stress-induced damaging effects. **(A–C)** Quantification of apoptotic cell fractions in 2D and 3D eMSCs exposed to oxidative stress **(A)**, heat shock **(B)**, and irradiation stress **(C)**; data are derived from the Annexin-V/DAPI flow cytometry assay performed 24 h **(A,C)**, or 3, and 24 h **(B)** after the stress insults. Data are shown as mean ± SD (*N* = 3). For early (AnV+/DAPI−) and late (AnV+/DAPI+) apoptotic cell fractions: ^∗^*p* < 0.05 vs. the control values (*t* = 0 h). **(D)** Flow cytometry assay for 3/7 caspase activation in 2D and 3D eMSCs (passage 8) exposed to heat shock reveals cell fraction with caspase activity in 3D sample (test is performed 6 h after cell heating). **(E)** Quantification of cell fraction with active caspase in 2D and 3D eMSCs after the heating. Data are normalized to the control values (*t* = 0 h) and shown as mean ± SD (*N* = 3). ^∗^*p* < 0.05 vs. the control. **(F)** Cell death in spheroids was confirmed by the propidium iodide staining performed 4 h after H_2_O_2_ (left) or heat treatments (right). Scale bar: 50 μm. Cntr, control cells; r.u., relative units; PI, propidium iodide.

In the next batch of experiments, we investigated the fate of oxidatively damaged cells and compared the viability of 2D and 3D eMSCs treated with H_2_O_2_. Annexin V assay showed that H_2_O_2_-damaged spheroid cells appeared to be more prone to the activation of apoptotic program than monolayer cells. One day after treatment of eMSC spheroids with non-cytotoxic for monolayer eMSCs doses of peroxide (100–200 μM), a substantial fraction of the late apoptotic (AnV+/DAPI+) cells was detected in spheroids (Figure 3A). Due to the protection of the inner layers of spheroids from the action of peroxide by the outer cells, the dose of H_2_O_2_ which is consumed by each outer cell in a sphere is higher than the dose per one monolayer cell. This fact may explain the increased level of apoptosis in spheroids observed after the peroxide exposure. To find out whether spheroid cells damaged by other stress insults activate the apoptotic program as well, we performed experiments with the heat shock (45°C, 30 min). Annexin V test carried out 24 h after cell heating indicated the presence of both late (AnV+/DAPI+) and early (AnV+/DAPI−) apoptotic cell fractions in 3D eMSC samples (Figure 3B). Moreover, the early apoptosis, revealed by both the Annexin V binding assay (see Figure 3B and flow cytometry histograms in Supplementary Figure S2) and tests for active 3/7 caspase (Figures 3D,E), was detected in 3D cells within a few hours after the heat shock (3–6h). In contrast to spheroid cells, tests for apoptosis performed at different time points after heating of the monolayer cells did not show any signs of apoptotic program activation. In addition to experiments with heat shock and peroxide, we performed a series of experiments with irradiation of cells. Similar to other stresses, exposure of spheroids to 10 Gy induced apoptosis, detected one day after the insult, while monolayer cells almost did not change their viability after irradiation (Figure 3C).

To check the distribution of the dead cells in the spheres exposed to different stresses, we stained H_2_O_2_-treated spheroids with a vital dye, propidium iodide (PI), and compare the staining pattern with that observed after the sphere heating (Figure 3F). In H_2_O_2_-exposed spheroids, dead cells were located only in the outer layers, whilst after the heat shock, dead cells were distributed evenly. This data additionally proves that 3D arrangement protects the inner cells of the spheres from the oxidative damage and death.

**FIGURE 4:**
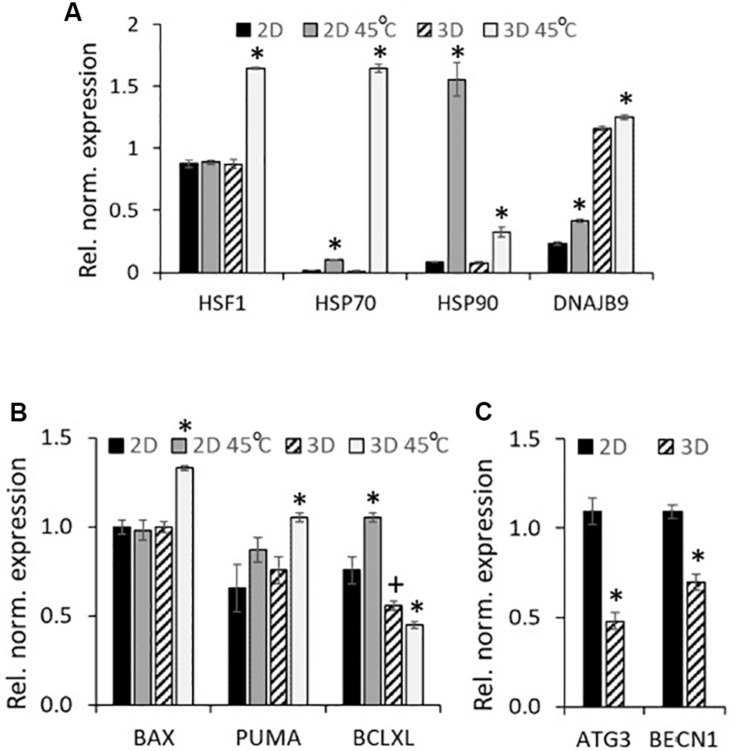
eMSC heat shock-induced gene expression profiles: upregulation of HSR genes (2D and 3D eMSCs), pro-apoptotic genes (3D eMSCs) and anti-apoptotic genes (2D eMSCs). **(A,B)** Expression of heat shock response **(A)**, as well as pro- and anti-apoptotic **(B)** genes in 2D and 3D eMSCs exposed to heating (3 h after the treatment). **(C)** Basal expression of autophagy-related genes in 2D and 3D eMSCs. Data **(A–C)** are shown as mean ± SD (*N* = 3). ^∗^*p* < 0.05 vs. the appropriate non-heated cells **(A,B)**, or vs. 2D eMSCs **(C)**. ^+^*p* < 0.05 vs. the 2D eMSC control **(B)**.

Next, with the purpose to analyze the reasons for the high tendency of spheroids to the activation of apoptosis program under stressors, we tracked the activation of the stress response programs in eMSC spheroids and eMSC monolayer after heating. We compared the expression of the genes responsible for the activation of HSR (Heat Shock Response): molecular chaperones HSP70 and HSP90, DNAJB9 [DnaJ Heat Shock Protein Family (HSP40) Member B9], as well as the transcription factor that activates the expression of chaperon genes, HSF1. RT-PCR analysis (Figure 4A) revealed strong upregulation of HSR genes 3 h after heating of both 2D and 3D cells, but the expression pattern in 2D and 3D eMSCs was different. A significant (about 100-fold) increase in HSP70 expression was observed in heated vs. control 3D eMSCs, accompanied by the induction of HSP70 key regulator, HSF1. In contrast, heated 2D eMSCs upregulated HSP90 gene. The expression level of DNAJB9, the co-chaperone involved in endoplasmic reticulum-associated degradation of misfolded proteins, increased slightly in both 2D and 3D eMSCs. Basing on these observations, we supposed that the reason for low survival rate of eMSCs in spheroids exposed to stress was not a deficiency of protective mechanisms, but a high capacity for activation of the apoptosis program. To check this point, we analyzed the levels of both heat-stress-induced and basal expression of genes related to apoptosis in 2D and 3D cultures. After the heat treatment, 3D eMSCs upregulated pro-apoptotic genes (Bax and PUMA) and down-regulated anti-apoptotic Bcl-xL gene, whilst 2D eMSCs upregulated Bcl-xL. In addition, we found low basal level of anti-apoptotic Bcl-xL gene expression in eMSC spheroids in comparison to monolayer cells (Figure 4B). As autophagy process is often considered as a protective mechanism that prevents cells from undergoing apoptosis, we checked the basal expression of autophagy-related (ATG3 and BECN1) genes, which appeared to be about 2 times lower in eMSC spheroids than in eMSC monolayer (Figure 4C).

Taken together, these findings point out to the high commitment of 3D MSCs to apoptosis activated by the cell damage caused by various stresses (thermal, oxidative stress and cell irradiation). The next part of our work was devoted to the analysis of 2D and 3D eMSC progeny cells, survived after stress administration.

### 2D eMSCs Respond to Stress by Activating the Stress-Induced Premature Senescence Program

**FIGURE 5:**
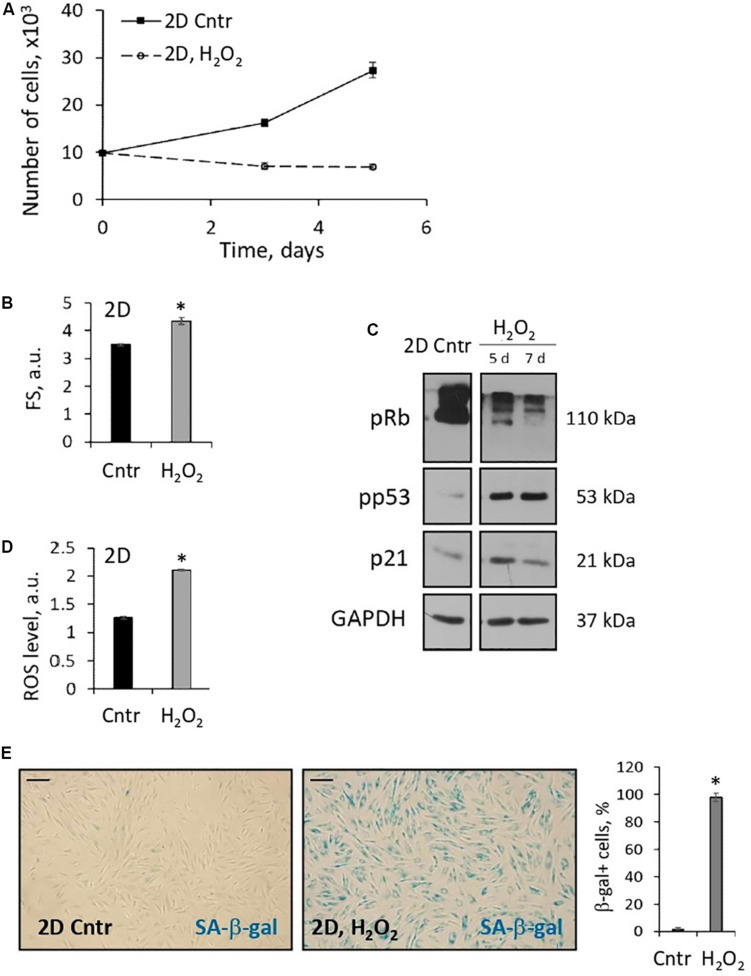
2D eMSCs respond to oxidative stress-induced damage by activation of premature senescence program. **(A)** Growth curves of H_2_O_2_-treated 2D eMSCs (passage 11) measured after the treatment. **(B)** Increase in the mean size of the H_2_O_2_-treated cells revealed by the forward scatter flow cytometry measurements (5 days after the treatment). **(D)** Increase of the ROS level in the H_2_O_2_-treated cells detected by flow cytometry analysis of H_2_DCFDA stained cells (5 days after the treatment). **(C)** Expression of the molecular markers of cell senescence revealed in H_2_O_2_-treated cells by Western blotting (5 and 7 days after the treatment). **(E)** Activity of SA-β-gal in H_2_O_2_-treated cells (5 days after the treatment): images and quantification of SA-β-gal + cell fraction. Data in panels **(A–D)** are shown as mean ± SD (*N* = 3). ^∗^*p* < 0.05 vs. the control cells. Scale bar: 100 μm. Cntr, control cells; a.u., arbitrary units; FS, forward scattering; ROS, reactive oxygen species; SA-β-gal, senescence associated β-galactosidase.

In our previous studies, we found that activation of the stress-induced premature senescence (SIPS) program is a typical response of 2D eMSCs to sublethal stresses (Alekseenko et al., 2014; Borodkina et al., 2014; Domnina et al., 2016; Kozhukharova et al., 2018; Kornienko et al., 2019). Here, we analyzed 2D eMSCs that survived incubation with 200 μM hydrogen peroxide (exposure causing apoptosis in 3D eMSC) and found signs of SIPS several days after H_2_O_2_ administration. As shown in Figure 5, H_2_O_2_-treated monolayer eMSCs stopped proliferating, as evidenced by cell growth curves. Moreover, flow cytometric analysis revealed in these cells a significant increase in the forward scatter signal (reflects the cell size), as well as in the ROS level measured using H_2_DCFDA probe. We also found in H_2_O_2_-treated eMSCs activation of the classical p53/p21/Rb signaling pathway that causes cell cycle arrest. Finally, the activity of senescence-associated β-galactosidase was detected 5 days after H_2_O_2_ exposure. Our findings indicate the development of premature senescence in monolayer cells induced by H_2_O_2_ treatment.

**FIGURE 6:**
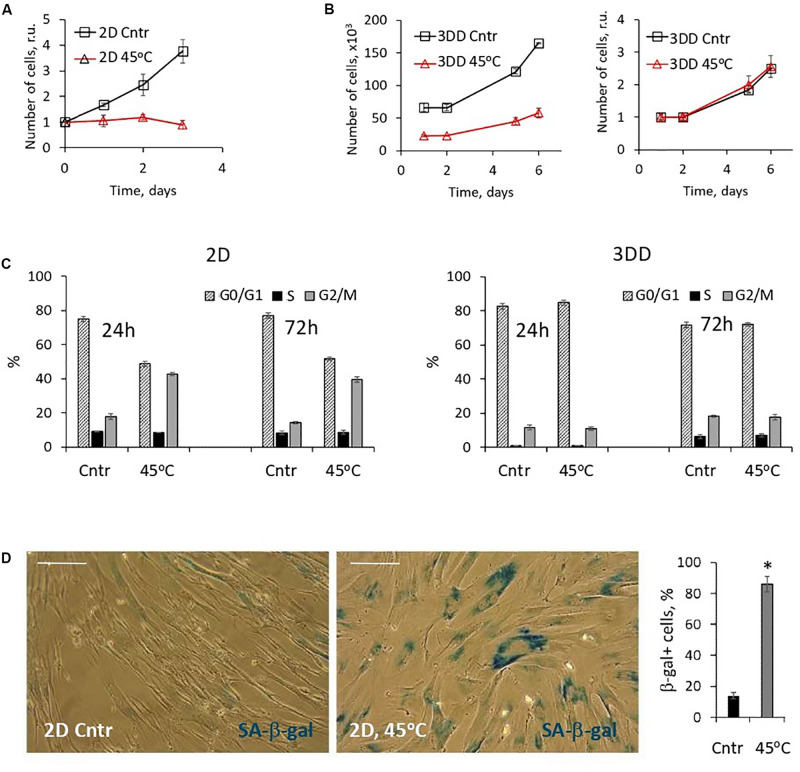
2D eMSCs, in contrast to 3D eMSCs, respond to the heat shock by activation of premature senescence program. **(A)** Growth curves of 2D eMSCs (passage 7) measured after the heat treatment. Data are normalized to the initial values. **(B)** Growth curves of 3DD eMSCs: cells were derived by trypsinization from the control spheroids and spheroids exposed to the heat shock, seeded as 2D culture, and after that counted daily. Curves reflect either the absolute number of cells in a dish (left), or relative numbers normalized to the initial values (right). Spheroids in this experiment were formed from eMSCs at passage 9. **(C)** Cell cycle phase distribution of 2D and 3DD eMSCs measured 24 and 72 h after the heat treatment. **(D)** Activity of SA-β-gal in 2D cells exposed to heating (5 days after the treatment): images and quantification of SA-β-gal + cell fraction. Data **(A–D)** are shown as mean ± SD (*N* = 3). Scale bar: 40 μm. Cntr, control cells; 3DD, 3D-derived cells; r.u., relative units; SA-β-gal, senescence associated β-galactosidase.

In addition to oxidative stress, we traced the fate of 2D cells that survived the heat shock. In contrast to the apoptotic reaction of 3D eMSC, heat shock induced a stop in the proliferation of damaged monolayer cells (Figure 6A), which was accompanied by a prolonged S-G_2_/M cell cycle block (Figure 6C) and expression of active senescence-associated β-galactosidase (Figure 6D). These results confirmed our previously published data and gave evidence about the induction of the SIPS program in 2D eMSC cultures that survived sublethal thermal stress (Alekseenko et al., 2014, 2018). To check whether 3D eMSCs that survived heat shock undergo stress-induced senescence, heated spheroids were dissociated 3 h after the stress, plated, and then were further cultured under adhesive monolayer conditions. Within a few days after the heat shock, we analyzed daily the distribution of the cell cycle phases and counted the number of 3D-derived eMSCs. Our experiments (Figure 6C) showed that one day after the stress, the descendants of both heated and control non-heated 3D eMSCs remained predominantly in the G_0_/G_1_ phase, in the state of the proliferation block which is typical for eMSCs cultured in spheroids (Domnina et al., 2018). At the same time, the amount of cells survived heating was significantly lower than the control values. Nevertheless, after several days, the survived descendants resumed division and did not differ in proliferation rates (Figure 6B) and cell cycle dynamics (Figure 6C) from the control 3D-derived eMSCs.

Thus, the results presented in this section show that eMSCs cultivated in monolayer respond to different types of stress by activating the SIPS program, while eMSCs cultivated in the configuration of spheroids are prone to stress-induced apoptosis, but not susceptible to the development of the premature senescence.

### Secretory Capacity of eMSCs Cultivated in Spheroids

**FIGURE 7:**
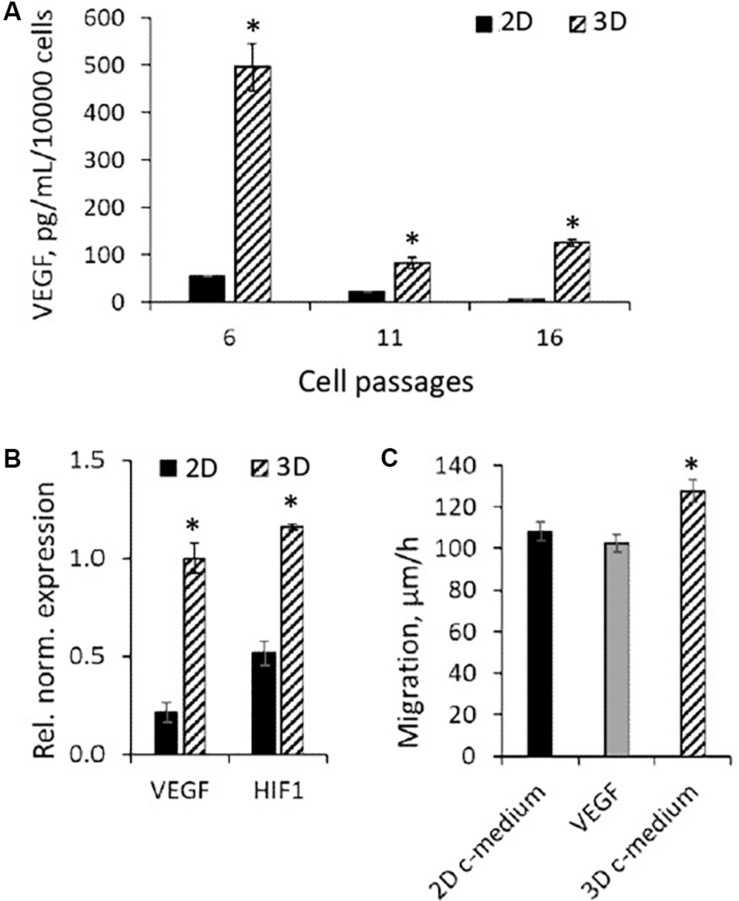
Secretory activity of 3D eMSCs. **(A)** VEGF level measured by ELISA in the growth medium of eMSC spheroids formed from eMSCs of different passages. **(B)** Upregulation of HIF1 and VEGF genes in spheroids in comparison to monolayer grown eMSCs (passage 8). **(C)** Rate of eMSC (passage 9) migration into the cell-free area in the dish with growth medium conditioned by 3D and 2D eMSCs (passage 8), as well as medium supplemented with 10 ng of recombinant human VEGF. Data **(A–C)** are shown as mean ± SD (*N* = 3). ^∗^*p* < 0.05 vs. 2D eMSCs. 2D c-medium, growth medium conditioned by 2D eMSCs; 3D c-medium, growth medium conditioned by 3D eMSCs; VEGF [in panel (C)], growth medium supplemented with 10 ng of VEGF.

One more specificity of eMSCs cultured in spheroids that can contribute to their high regenerative potential may be the shift in the cell secretory phenotype. In our previous work, we showed that cultivation in spheroids increases the expression of anti-inflammatory eMSC genes (Domnina et al., 2018). At the same time, the literature data indicates that in the case of MSCs from adipose tissue, bone marrow, and umbilical cord blood cultivated in spheroids, the secretion of angiogenic factors, such as vascular endothelial growth factor (VEGF), also increases (Madrigal et al., 2014). Using enzyme-linked immunosorbent assay (ELISA), we compared the basal level of VEGF-A secretion in eMSCs at different passages seeded both in3D and 2D configuration. In 2D eMSCs, VEGF secretion has been significantly reduced after cell passaging; however, 72-h culturing of eMSC in 3D structures markedly increased VEGF secretion at each cell passage (Figure 7A).

Vascular endothelial growth factor is known to be a cytokine whose secretion increases with hypoxia. Using the RT-PCR, we found that the high level of VEGF secretion in eMSC spheroids was accompanied by enhanced expression of the VEGF-A genes and the hypoxia-inducible factor HIF-1α, which controls the transcriptional activity of VEGF (Figure 7B).

To investigate the effect of soluble factors secreted by 3D eMSCs on wound healing, we carried out a scratch assay, using condition medium from spheroids and monolayer as potential stimulators of cell migration (Figure 7C). The medium obtained from spheroids significantly increased the rate of scratch healing in comparison with 2D eMSCs, which indicates the stimulating effect of spheroid-secreted paracrine factors on cell motility during wound closure. At the same time, the addition of exogenous recombinant VEGF to the 2D eMSC medium did not enhance cell migration that points out to the complex character of the eMSC wound healing potency.

## Discussion

When developing cell technologies for the correction of various human pathologies, it is worth taking into account that cell therapeutic potential is determined by a whole range of functional and molecular characteristics of cells. This includes immune tolerance, resistance to stress, which prevents cell death during transplantation, the ability to secrete specific factors necessary for the treatment of pathologies, etc. Each of these characteristics deserves a detailed study for each type of pathology and type of transplanted material. In this article, using eMSCs as an example, we have proven the ability of these cells, cultured as spheroids and as monolayer, to correct defects that are non-specific for eMSC tissue origin – skin wounds. The therapeutic potential of spheroids turned out to be higher than that of cells cultured in a monolayer. By analyzing the possible causes of high therapeutic potency of eMSC spheroids, we compared the resistance of 3D and 2D eMSCs to the cytotoxic effect of stress insults, and evaluated the secretory properties of eMSCs cultivated in 2D and 3D cell environment.

In a comparative analysis of the 3D and 2D eMSC resistance to stress, we investigated cell response to different types of damaging effects. First, we studied the oxidative stress induced by exogenous hydrogen peroxide. H_2_O_2_ is a source of local oxidative stress that accompanies the process of inflammation in the regenerating tissues of the body. Our *ex vivo* experiments have shown that the outer cell layers in spheroids shield the damaging effects of exogenous H_2_O_2_. The antioxidant system of these cells effectively eliminates H_2_O_2_ and serves as a protective barrier that leaves the inner layers of cells intact. As a result, the number of cells with DNA damage in H_2_O_2_-treated spheroids is lower than that in the monolayer culture exposed to peroxide. It is worth noting that, in addition to the screening effect, the low level of DNA damage in spheroids observed in our experiments can be explained by the low proliferative index of 3D cells. Our analysis of the 3D eMSC cell cycle (Domnina et al., 2018) showed that cells in spheroids do not proliferate that means they are less susceptible to the DNA-damaging stress factors. In contrast to eMSCs from spheroids, actively proliferating monolayer cells are more sensitive to the genotoxic effect of stress (Alekseenko et al., 2018).

Further, we investigated the fate of cells damaged by oxidative stress. It turned out that under the same H_2_O_2_-treatment conditions, damaged cells of the outer layer of spheroids activated the apoptosis program and died, while 2D cells with damaged DNA survived. In addition, we compared the viability of 3D and 2D eMSCs after exposure to heat shock and irradiation. In all cases, stress led to the emergence of a high percentage of apoptotic cells in spheroids, while no apoptosis was detected in 2D eMSCs. To find out, whether the higher sensitivity of spheroid cells to stress can be an attribute of only eMSCs, we conducted experiments with 2D and 3D adipose MSCs and obtained similar results (not shown here): heat shock induced apoptosis in 3D MSCs, but did not lead to the death of monolayer cells.

Analysis of the HSR gene expression revealed significant upregulation of the chaperone genes in both 3D and 2D eMSCs after the heat stress, which demonstrates the high efficiency of the cell defense molecular mechanisms. Interestingly, the pattern of HSR gene expression in 3D and 2D eMSCs was different, which indicates diverse pathways of the protective response to stress.

Taken together, these data suggest that stress-induced death of cells in eMSC spheroids is not due to the deficiency of protective mechanisms, but due to the high commitment of 3D eMSCs to apoptosis. This assumption is further confirmed by the low basal expression of anti-apoptotic gene Bcl-xL and autophagy-controlling genes (ATG3 and BECN1). It is well known that upon activation of apoptosis pathways in cells, an autophagy program that performs a cytoprotective function is often suppressed (Gordy and He, 2012), and we suppose that basal suppression of autophagy-controlling genes in 3D eMSCs may evidence about the predisposition to apoptosis even under the normal physiological conditions. It should be noted that our data are in a good agreement with the results obtained for cell spheroids consisting of the other types of MSCs (Bartosh et al., 2013). According to these studies, caspase activity observed in MSC spheroids stimulates the secretion of interleukins, which determines their anti-inflammatory properties and is thus one of the key fundamental properties of 3D MSCs that affect their therapeutic potential.

Next, our studies have shown, that in contrast to the apoptosis program, which is activated in damaged 3D eMSCs, a program of premature senescence of cells is initiated in 2D eMSC cultures exposed to stress. It is known that cells of different origin can respond differently to stressful insults. We previously showed that stress induces premature senescence in mesenchymal stem cells, but apoptosis in embryonic stem cells (Alekseenko et al., 2012, 2014). The results obtained in this work prove that 3D cultivation is able to switch the programs of eMSC stress response, cancel the activation of senescence pathways and activate the apoptotic program. We suggest that this effect can be associated with phenotypic shift, which is accompanied by the enhanced expression of the key pluripotent transcription factors Nanog and Sox observed in 3D cultured eMSC (Domnina et al., 2018). Summarizing our studies on the stress response of 3D eMSCs, in spite of the shielding effect of the outer cell layers in spheroids against some types of the cell damage (such as external oxidative stress), spheroid therapeutic potency is not related to maintaining high cell viability in case of damage during transplantation. Instead, we conclude that predisposition to apoptosis provides the programmed elimination of damaged cells that may contribute to the therapeutic efficiency and transplant safety of spheroids. Recently, it has been shown that damaged cells undergoing SIPS can be less effective and even dangerous in clinical applications. Senescent cells possess attenuated angiogenic potential (Ratushnyy et al., 2020), and when injected into an organism for the therapeutic needs, can induce inflammation and oncological transformation of healthy tissues due to the potentially harmful secretory phenotype (Turinetto et al., 2016).

In contrast, secretome of 3D-cultured cells can be considered in itself as a potent therapeutic agent. In our previous works on the use of eMSC spheroids for the treatment of infertility, we found that 3D cultivation of eMSCs significantly increases the expression of genes responsible for the anti-inflammatory factors secretion (Domnina et al., 2018). In the present work, we compared the ability of eMSCs cultured in 3D and 2D geometry to secrete VEGF, an endothelial vascular growth factor with angiogenic, trophic, and anti-apoptotic properties. We found that similar to spheroids from MSCs of adipose tissue, bone marrow, and umbilical cord blood, eMSC spheroids increase the level of VEGF secretion ten-fold compared to the eMSC monolayer. Using *in vitro* model of wound healing (scratch assay), we have shown that administration of conditioned 3D eMSC medium increases cell mobility and the rate of scratch closure to a greater extent than conditioned 2D eMSC medium, proving that enhanced secretory activity can promote wound healing potential of eMSC spheroids. At the same time, supplementation the culture medium with recombinant VEGF did not increase cell mobility that proves that high therapeutic potency of 3D cells is not due to high VEGF secretion only but instead is determined by the full repertoire of released molecules (such as chemokines, growth factors, miRNAs, etc.).

## Conclusion

Transplantation of human eMSCs cultured as spheroids or as a monolayer into rats contributes to the treatment of skin wounds – the defects that are non-specific for the tissue origin of eMSCs. The therapeutic effect of eMSCs from spheroids exceeds the effect of eMSCs cultured in the monolayer configuration. In spite of the shielding effect of the outer cell layers in spheroids against some types of the cell damage (such as external oxidative stress), spheroid therapeutic potency is not related to maintaining high cell viability in case of damaging during transplantation. Cells in spheroids respond to damage induced by various stress insults by activating the apoptosis program, in contrast to monolayer cells in which a program of premature senescence is induced. In the *in vitro* wound healing model, administration of the conditioned 3D eMSC medium increases cell mobility and the rate of scratch closure to a greater extent than conditioned 2D eMSC medium, proving that enhanced secretory activity can promote wound healing potential of eMSC spheroids. Thus, cultivation in 3D cell environment alters eMSC vital programs and therapeutic efficacy.

## Materials and Methods

### Cell Cultures

Human endometrial mesenchymal stem cell lines (eMSCs) were derived from a desquamated endometrium of menstrual blood from healthy donors (Zemel’ko et al., 2011). Endometrial MSCs express CD13, CD29, CD44, CD73, CD90, CD146, and CD105 surface markers and are negative for the hematopoietic markers CD34 and CD45, they possess the ability to differentiate into adipocytes, chondrocytes and osteoblasts (Zemel’ko et al., 2011). Cells were cultivated in DMEM/F12 growth medium (Gibco) containing 10% fetal bovine serum (FBS, HyClone), 1% L-glutamine (Gibco) and 1% penicillin-streptomycin (Gibco). Endometrial MSCs were maintained at 37°C in a humidified chamber with 5% CO_2_ and subcultured twice per week. In this study we used cells from 3 to 12 passage. The experiments were approved by the Ethics Committee of the Almazov National Medical Research Centre (Saint Petersburg, Russia) and performed in accordance with the institutional guidelines. All cell donors signed an informed consent for voluntary participation.

### Spheroid Formation

Spheroids (3D cultures) were formed from monolayer eMSCs (2D cultures) using the hanging drop technique. Seven thousand cells per 35 μL of DMEM/F-12 medium containing 10% of FBS were placed in drops on the cover of 10-cm Petri dishes (Corning) and inverted. During the next 48 h, cells spontaneously aggregated in hanging drops and formed spheres, which were transferred then to dishes coated with 2-hydroxyethyl methacrylate (HEMA, Sigma) and cultured in 2 ml of full growth medium for 24 h.

### Cell Treatments

Within this study cells were subjected to different stress insults – oxidative, thermal, and cell irradiation stresses. In each experiment, the cell count was equal (100,000 cells maintained as 2D or 3D culture in 2 ml of medium per 3 cm-diameter Petri dish). Oxidative stress was induced by incubation of eMSCs with hydrogen peroxide solution in the FBS free medium. To perform the heat shock experiments, dishes with 2D and 3D eMSC cultures were incubated at 45°C for 30 min in the water bath. For cell irradiation experiments eMSCs were exposed to 10 Gy doses of irradiation using a stationary X-ray device (0.49 Gy/min).

### Live Cell Microscopy

Two-dimensional eMSCs and already formed eMSC spheroids, which both expressed genetically encoded biosensor of hydrogen peroxide HyPer, were placed in 4-well chambered coverglass plate (Thermo Fisher Scientific) with 750 μl of full growth medium per well. Cells were left overnight to ensure either the formation of monolayer (in the case of 2D cultures), or attachment and immobility of spheroids (in the case of 3D cultures). On the next day before the experiment, the complete medium was changed to serum-free DMEM/F-12, and the plate was placed to the confocal microscopy chamber (Olympus FV3000) maintained at 37°C. Two-hundred microliter of hydrogen peroxide was then added to each well and the consecutive shots in 405 nm and 488 nm channel were taken each 5 min. The obtained images were then processed using ImageJ software as described in Mishina et al. (2013). Image processing yields a ratiometric HyPer signal that visualize the hydrogen peroxide concentration profile inside individual cell. The similar image processing was applied for 2D eMSCs. Visualization of the dead cells in the spheres exposed to oxidative stress or heat treatment was achieved using PI staining (40 μg/ml, 20 min at 37°C) performed 4 h after the stress insults.

### Apoptosis Assay

For detection of apoptosis in cells exposed to stress insults, 3, 6, or 24 h after the treatments both 2D and 3D cell cultures were trypsinized, suspended in the buffer solution and double-stained with 4′,6-diamidino-2-phenylindole (DAPI) and Annexin V Alexa Fluor 647 conjugate (Invitrogen^TM^) according to the manufacturer’s instructions. Cell samples were analyzed with CytoFLEX flow cytometer (Beckman Coulter, 405/633 nm lasers).

For detection of the caspase activity, 6 h after the treatments 2D and 3D cells were trypsinized, suspended in the growth medium and stained with CellEvent Caspase-3/7 Green Detection Reagent (Invitrogen^TM^) for 20 min at 37°C. After the staining procedure, cell samples were analyzed with CytoFLEX flow cytometer (Beckman Coulter, 488 nm laser).

### Cell Proliferation Assay

For analysis of the proliferative activity of cells exposed to stress insults, 2 h after the treatments, 2D and 3D cultures were trypsinized and plated as adherent cultures. Cells derived from the spheres are referred to as 3D-derived (3DD) cells. After seeding, once a day, 2D and 3DD cells were detached, counted, permeabilized with 0.1% Triton X-100 and stained for 5 min with 2 μg/ml of DAPI to perform the cell cycle analysis. Cell cycle phase distribution was measured with CytoFLEX S flow cytometer (Beckman Coulter, 375 nm laser).

### DNA Damage Assay

One hour after H_2_O_2_ exposure, 2D and 3D cultures were trypsinized, washed twice with PBS, and then fixed/permeabilized using Nuclear Factor Fix and Perm Buffer Set (BioLegend). For specific detection of γH2AX foci accumulation, cells were incubated with anti-γH2AX antibodies (1:200, Abcam) for 1 h at room temperature in the dark. After washing with PBS, cells were incubated with 1:500 solution of goat-anti-mouse (GAM) Alexa Fluor 488 secondary antibodies and 1 μg/ml of DAPI for 30 min at room temperature in the dark. The γH2AX foci accumulation, as well as its distribution among the cell cycle phases, were then analyzed with CytoFLEX flow cytometer (Beckman Coulter, 405/488 nm lasers).

### *In vivo* Wound Healing Assay

Twelve adult albino male Wistar rats with 230–250 g weight were used in experiments. The animals were maintained in the designated animal care facility with free access to tap water and food. All manipulations were done under aseptic conditions. Animals were anesthetized by intramuscular injection of Zoletil 100 (Virbac) in dose 5 mg/kg weight. The animal backs were shaved and disinfected with 70% alcohol. Three pieces of full-thickness skin (1.5 cm × 1.5 cm) on central side of the back were excised to create three skin wounds. Immediately after, monolayer eMSC in suspension (4 × 10^6^ cells/per wound) and eMSC spheroids (4 × 10^6^ cells in total/per wound) were implanted in 100 μl PBS solution around the prepared full-thickness skin wounds. The same volume of PBS without cells was used for the control wound. Finally, each animal was covered with bandage. On the day of surgery and every day thereafter the open wounds were photographed. The wound area was measured by tracing the wound margin and calculating pixel area using the image analyzer ImageJ (Rasband, 2012). The time of wound closure was defined as the time at which the wound bed was completely reepithelialized. The percentage of wound closure was calculated as follows: (area of original wound - area of actual wound)/area of original wound × 100% (Santos et al., 2015). A wound was considered completely closed when wound area was equal to zero. Some rats were sacrificed 7 days after surgery for histological examination. Collected skin tissue was fixed in 10% buffered formalin for 24 h and embedded in paraffin. The tissue blocks were cut into 5-mm-thickness sections followed by routine hematoxylin and eosin staining. Epithelialization (length of the regenerating epithelia) and granulation tissue thickness were assessed by the light microscopy.

All experimental procedures with animals were performed according to the Institutional guidelines for the care and use of laboratory animals. All studies on animals were performed after approval by the Institutional Animal Care and Use Committee of Institute of Cytology RAS (Assurance Identification number F18-00380).

### *In vitro* Wound Healing Assay

For *in vitro* wound healing assay a standard *in vitro* technique for probing collective cell migration was used according to the recommendations (Jonkman et al., 2014).

We compared the dynamics of the migration into the cell-free area of 2D eMSCs, treated with culture medium, containing 1% FBS and 10 ng recombinant human VEGF (Gibco Life Technologies, United States) or conditioned growth medium from 2D and 3D eMSCs, containing 1% FBS. Two-well silicone insert (Ibidi) in a 24-well plates were used for gap creation. Twenty-five thousand cells in growth medium were seeded on both sides of the insert and incubated overnight after a confluent monolayer was formed. The next day inserts were removed, cells were washed and compared samples of medium were added to the wells. Photographs of the cells were taken at 0, 8 and 24 h to monitor the gap close. The gap size was measured as a function of time using Fiji/ImageJ software package.

### Enzyme Linked Immunosorbent Assay

The concentration of VEGF in the cell medium was measured using ELISA kit DuoSet Human VEGF (R&D-system, United States) following the manufacturer’s instructions. Quantification was performed by building a standard curve using known concentrations of human VEGF. Cell medium was collected from the same number of 2D and 3D cells incubated for 3 days in the growth medium with 1% FBS.

### Detection of Cell Senescence

Three to five days after the stress insults, cells that express senescence associated β-galactosidase were detected in 2D cultures with the use of senescence β-galactosidase staining kit (Cell Signaling Technology, United States) according to the manufacturer’s instructions. For each experimental point not less than 100 randomly selected cells were analyzed. Besides, the increase in the cell size and reactive oxygen species (ROS) production in the senescent cell cultures was assessed. For this purpose, 2D cells were incubated in the 5 μM staining solution of ROS-sensitive probe 2′,7′-dichlorodihydrofluorescein diacetate (H2DCFDA, Invitrogen, D399) in the serum-free medium for 30 min in the dark at 37°C. After that, cells were harvested, suspended in the fresh medium and immediately analyzed with CytoFLEX flow cytometer (Beckman Coulter, 488 nm laser). To evaluate the increase in the cell size which accompanies the senescence, forward scatter signal was monitored in the parallel experiments.

### Western Blotting

Western blotting was performed as described previously (Borodkina et al., 2014). SDS-PAGE electrophoresis, transfer to nitrocellulose membrane and immunoblotting with ECL (Thermo Fisher Scientific, United States) detection were performed according to standard manufacturer’s protocols (Bio-Rad Laboratories, United States). Antibodies against the following proteins were used: glyceraldehyde-3-phosphate dehydrogenase (GAPDH) (clone 14C10) (1:1000, #2118, Cell Signaling Technology, United States), phospho-p53 (Ser15) (clone 16G8) (1:700, #9286, Cell Signaling Technology, United States), p21Waf1/Cip1 (clone 12D1) (1:1000, #2947, Cell Signaling Technology, United States), phospho-Rb (Ser 807/811) (1:1000, #8516, Cell Signaling Technology, United States), well as horseradish peroxidase-conjugated goat anti-rabbit IG (GAR-HRP, Cell Signaling Technology, United States) (1:10000).

### qRT-PCR Assays

To analyze gene expression, total RNA was isolated with RNeasy Micro Kit (Qiagen) according to the manufacturer’s instructions. RNA concentration was quantified using NanoDrop ND-1000 Spectrophotometer (NanoDrop Technologies, Inc, Wilmington, DE, United States). cDNA was obtained by reverse transcription of 500 ng RNA using the RevertAid H Minus First Strand cDNA Synthesis Kit (Fermentas) according to the manufacturer’s instructions. It was subsequently amplified with specific primers, using DreamTaq^TM^ PCR Master Mix (2X) (Thermo Fisher Scientific) with Cyclo Temp Amplificator. cDNA was amplified with specific primers, using EvaGreen^®^ dye (Biotium) and DreamTaq^TM^ PCR Master Mix (2X) (Thermo Fisher Scientific) in the BioRad CFX-96 real time system (BioRad, CA, United States), according to the kit’s enclosed protocol. The volume of PCR reactions was 20 μl. Expression of target genes was normalized to GAPDH gene. Primers and reaction conditions are presented in the Table 1. All amplification reactions were performed in triplicates. Each gene expression was calculated using Real-Time PCR Analysis Software BioRad, CA, United States. Experiments were repeated at least three times.

**TABLE 1:** The primers and conditions for qRT-PCR.

Symbol	Primer sequence	Amplification conditions	PCR product size (bp)	NCBI Reference Sequence
VEGF	F: 5′-CTACCTCCACCATGCCAAGT-3′	93°C, 20 s, 59°C, 20 s, 72°C, 30 s	95	NM_001025366.3
	R: 5′-GATAGACATCCATGAACTTCACCA-3′			
ATG3	F: 5′-GTGGCAGCGAGGACATTTTC-3′	93°C, 20 s, 60°C, 20 s, 72°C, 30 s	250	NM_001278712.2
	R: 5′-CCATGTTGGACAGTGGTGGA-3′			
Becn1	F: 5′-GGCTGAGAGACTGGATCAGG-3′	93°C, 20 s, 60°C, 20 s, 72°C, 30 s	127	NM_001313998.2
	R: 5′-CTGCGTCTGGGCATAACG-3′			
HIF1	F: 5′-CCGCCCGCTTCTCTCTAGT-3′	93°C, 20 s, 60°C, 20 s72°C 30 s	240	NM_181054.3
	R: 5′-TGGCTGCATCTCGAGACTTT-3′			
HSF1	F: 5′-CAGCTGATGAAGGGGAAGCA-3′	93°C, 20 s, 60°C, 20 s, 72°C, 30 s	216	NM_005526.4
	R: 5′-ACTGTCGTTCAGCATCAGGG-3′			
HSP70	F: 5′-ATGCGGCCAAGAACCAGGTG-3′	93°C, 20 s, 61°C, 20 s, 72°C, 30 s	307	NM_005345.5
	R: 5′-GCGCTGCGAGTCGTTGAAGT-3′			
HSP90	F: 5′-AATCGGAAGAAGCTTTCAGA-3′	93°C, 20 s, 55°C, 20 s, 72°C, 30 s	446	NM_005348.3
	R: 5′-GTGCTTGTGACAATACAGCA-3′			
HSP40	F: 5′-AGTCGGAGGGTGCAGGATATT-3′	93°C, 20 s, 60°C, 20 s, 72°C, 30 s	153	NM_012328.3
	R: 5′-TTGATTTGGCGCTCTGATGC-3′			
BclXl	F: 5′-GCTTGGATGGCCACTTACCT-3′	93°C, 20 s, 60°C, 20 s, 72°C, 30 s	231	NM_001317919.2
	AS: 5′-GGGAGGGTAGAGTGGATGGT-3′			
PUMA	F: 5′-GAC CTC AAC GCA CAG TAC GA-3′	93°C, 20 s, 60°C, 20 s, 72°C, 30 s	147	XM_006723141.3
	R: 5′-CAC CTA ATT GGG CTC CAT CT-3′			
BAX	F: 5′-GGG TTG TCG CCC TTT TCT-3′	93°C, 20 s, 60°C, 20 s, 72°C, 30 s	91	NM_138763.4
	R: 5′-CAG CCC ATG ATG GTT CTG ATC AG-3′			
GAPDH	F: 5′-GAGGTCAATGAAGGGGTCAT-3′	93°C, 20 s, 60°C, 20 s, 72°C, 30 s	100	NM_001357943.2
	R: 5′-AGTCAACGGATTTGGTCGTA-3′			

### Statistical Analysis

All data are presented as the mean values of at least three independent experiments with standard deviations. Statistical significance was calculated using either ANOVA-Tukey test in case of multiple comparisons or Student’s *t*-test in case of pair comparisons. *p*-values < 0.05 were considered significant.

## Data Availability Statement

All datasets generated for this study are included in the article/Supplementary Material.

## Ethics Statement

The experiments were approved by the Ethics Committee of the Almazov National Medical Research Centre (Saint Petersburg, Russia) and performed in accordance with the institutional guidelines. All cell donors signed an informed consent for voluntary participation.

## Author Contributions

JI, AD, LA, IK, AB, IF, and OL wrote the main manuscript text and prepared figures. JI, AD, LA, IK, AB, IF, IS, NP, and OL performed experimental part of the work and analyzed data. NN and IF supervised the work. All authors reviewed the manuscript.

## Conflict of Interest

The authors declare that the research was conducted in the absence of any commercial or financial relationships that could be construed as a potential conflict of interest.
