# Supplementary material for: Three-Dimensional Compaction Switches Stress Response Programs and Enhances Therapeutic Efficacy of Endometrial Mesenchymal Stem/Stromal Cells
Source: Front Cell Dev Biol. 2020 Jun 16;8:473. doi: 10.3389/fcell.2020.00473 (PMC7308716; doi:10.3389/fcell.2020.00473)
Supplement: Supplementary file 1 [file Image_1.PDF]

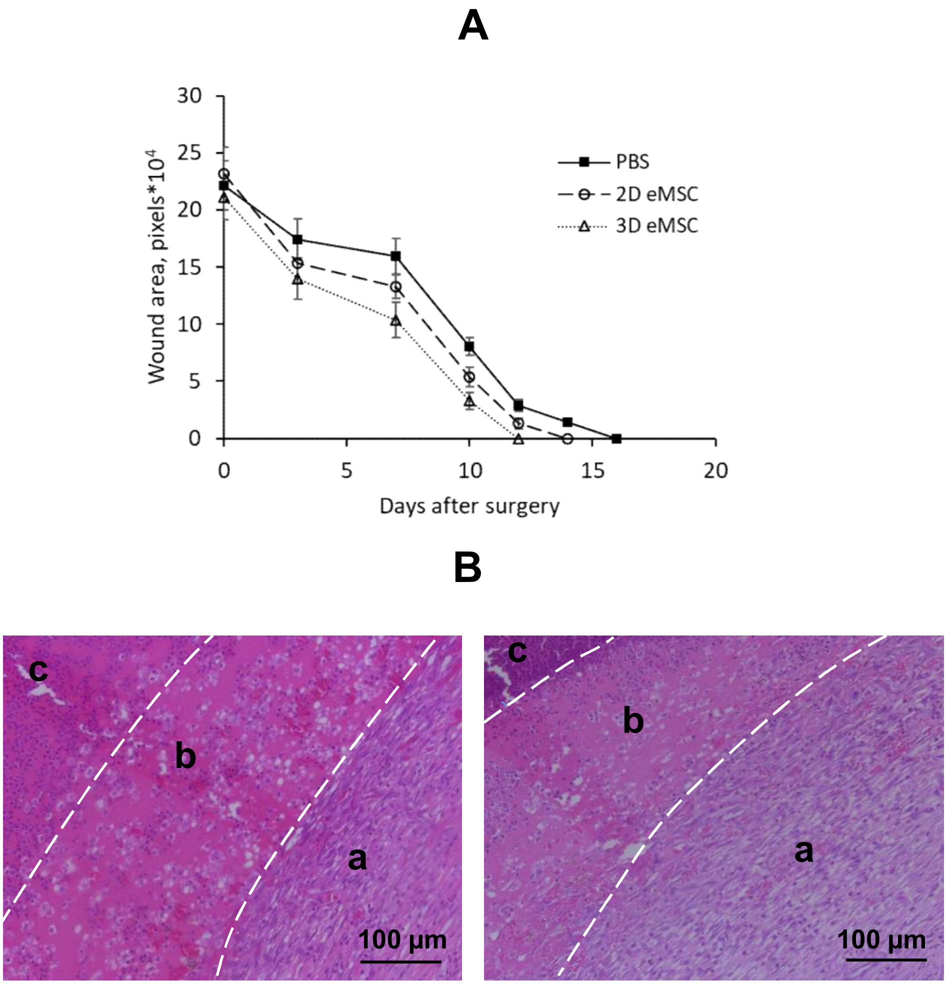

Figure 1S. Wound closure in rat after 2D and 3D eMSC transplantation.
(A) The wound size dynamics derived from the calculation of the pixel area of the wounds. Data are shown as mean ± SD (N = 4).
(B) Histological slices stained by hematoxylin and eosin at day 7 after the transplantation of 2D (left image) and 3D (right image) eMSCs, a- granulation tissue, b – epidermis, c - scab consisting of fibrin with leukocyte infiltration; 200x magnification.

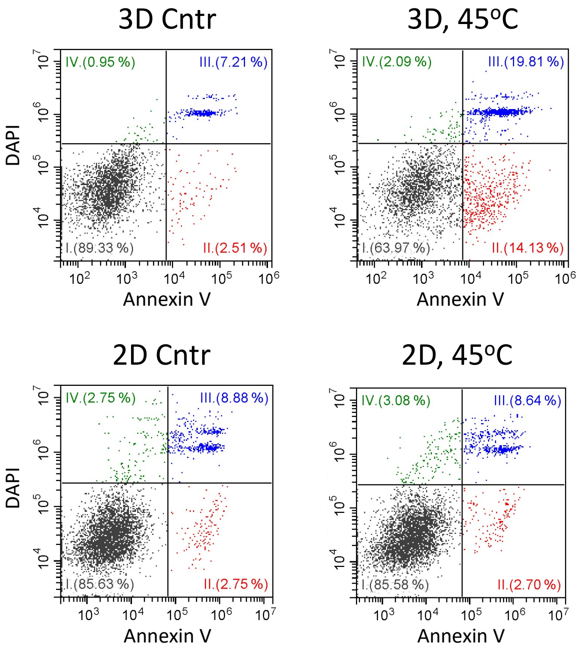

Figure 2S. Apoptosis assay of 2D and 3D eMSCs exposed to heat shock. Annexin-V/DAPI flow cytometry histograms measured in 2D and 3D eMSCs (passage 8) exposed to heat shock (test was performed 6 hours after cell heating). Colour marking: black, viable AnV-/DAPI- cells; red, early apoptotic AnV+/DAPI- cell fraction; blue, late apoptotic AnV+/DAPI+ cell fraction; green, non-apoptotic AnV-/DAPI- dead cells.
